# P291: Risk assessment in hemodialysis practice in universitary hospital center of Abidjan Cocody

**DOI:** 10.1186/2047-2994-2-S1-P291

**Published:** 2013-06-20

**Authors:** J Sissoko, R-H Oyourou

**Affiliations:** 1 SAMU-CI, Great Burned Center, Abidjan, Côte d'Ivoire

## Introduction

The practice of hemodialysis is a complex and technical high risk of infection to both the patient and the caregiver. Indeed, the infection is a major cause of morbidity and mortality in renal dialysis (15% of deaths).

## Objectives

To evaluate the practices of hemodialysis center staff CHU Cocody.

## Methods

This is a cross-sectional study during the month of February 2012 with the health personnel in the hemodialysis center. Mainly nurses were interviewed. This study focused on the observation of indications for hand hygiene, compliance with asepsis, environmental control and risk prevention methods used.

## Results

Almost all (96%) of the staff interviewed remove the jewelry on the hands and wrists before work. The equipment needed for dialysis is placed on a trolley that is regularly disinfected in 65% of cases. With regard to hand hygiene, hydroalcoholic solution is not available. After simple washing of hands, the staff uses alcohol for disinfection (55%) before wearing gloves. However, before going to another patient, staff dons new gloves. Disinfection is made one time (100%) and not four times. The arms and hands of patient with FAV are not washed before the meeting. Only the site is disinfected with alcohol before injection of the anticoagulant FDI. When connecting and disconnecting the generator, the gloves are also removed before further handling (83%). The screen is cleaned without being disinfected. The means of protection available at the Cocody Hemodialysis Center are essentially non-sterile gloves (80%) and sterile gloves (47%). Availability of masks is rare (5%); gowns and safety glasses are non-existent.

## Conclusion

This study identified the risk of infection in the Cocody hemodialysis center. The priority is mainly the provision of equipment necessary for patient safety.

## Competing interests

None declared

